# How can we meet the information needs of patients with early stage papillary thyroid cancer considering radioactive iodine remnant ablation?

**DOI:** 10.1111/j.1365-2265.2010.03966.x

**Published:** 2011-04

**Authors:** A M Sawka, S Straus, A Gafni, J D Brierley, R W Tsang, L Rotstein, S Ezzat, L Thabane, G Rodin, S Meiyappan, D David, D P Goldstein

**Affiliations:** * Division of Endocrinology, Department of Medicine, University Health NetworkToronto; † Divisions of Endocrinology, Department of Medicine, University of TorontoToronto; ‡ Divisions of Geriatrics and Internal Medicine, Department of Medicine, University of TorontoToronto; § Department of Clinical Epidemiology, McMaster UniversityHamilton; ¶ Departments of Radiation Oncology, University Health Network and the University of TorontoToronto, ON, Canada; ** Departments of Surgery, University Health Network and the University of TorontoToronto, ON, Canada; †† Departments of Psychosocial Oncology, University Health Network and the University of TorontoToronto, ON, Canada; ‡‡ Departments of Otolaryngology, University Health Network and the University of TorontoToronto, ON, Canada

## Abstract

In patients with early stage papillary thyroid carcinoma (PTC) who have had a thyroidectomy, the decision must be made to accept or reject radioactive iodine remnant ablation (RRA). Counselling patients about this decision can be challenging, given the medical evidence uncertainties and the complexity of related information. Although physicians are the primary source of medical information for patients considering RRA, some patients have a desire for supplemental information from sources such as the internet. Yet, thyroid cancer resources on the internet are of variable quality, and some may not be applicable to the individual case. We have developed a computerized educational tool [called a decision aid (DA)], directed to patients with early stage papillary thyroid cancer, and intended as an adjunct to physician counselling, to relay evidence-based medical information on disease prognosis and the choice to accept or reject RRA. DAs are tools used to inform patients about available treatment options and have been utilized in oncologic decision-making. We tested our web-based DA in fifty patients with early stage PTC and found that it improved medical knowledge. Furthermore, participants found the technical usability of the tool acceptable. We are currently conducting a randomized controlled trial comparing the use of the DA plus usual care to usual care alone to confirm the educational benefit of the website and examine its impact on the decision-making process. In the future, DAs may play an expanded role as an adjunct to physician counselling in the care of patients with thyroid cancer.

## Introduction

Thyroid carcinoma is increasingly being diagnosed^1^–^5^, and the most common subtype is papillary thyroid carcinoma (PTC).^1^,^6^ New cases of PTC are most commonly diagnosed at an early stage, with a low risk of disease-related mortality.^7^ After the standard surgical removal of the thyroid (total or near-total thyroidectomy), radioactive iodine remnant ablation (RRA) treatment or no RRA (with close medical follow-up) is considered acceptable options for management of early stage PTC, with some flexibility in the approach depending on the individual case characteristics and treatment preferences.^8^

## Information needs of patients with thyroid cancer considering RRA

Decision-making about accepting or rejecting RRA for early stage PTC is challenging, as there are no published randomized controlled trials testing the benefit of this intervention on long-term thyroid cancer outcomes, and results from existing observational medical evidence are conflicting.^9^ In a recent qualitative study, thyroid cancer survivors identified some key medical information needs related to the consideration of RRA (or no RRA), and these include the following: information on the disease prognosis, clearly delineated treatment options (RRA or no RRA, with follow-up implications), the treatment rationale, the potential risks and benefits of the treatment options and related medical evidence uncertainties, current clinical practice guideline recommendations and reproductive considerations (for some patients) (Table 1).^10^ The desired level of detail of medical information and the degree of involvement in medical decision-making is highly variable among patients with thyroid cancer, with the spectrum spanning from the preference for basic medical information and leaving decisions entirely to the physician, to the preference of highly detailed information and active participation in decision-making.^10^ The information needs of some patients with thyroid cancer may not be fully met in their clinical encounter with their healthcare providers.^10^ For patients with thyroid cancer desiring additional medical information to supplement physician counselling, the internet is easily accessible resource, but unfortunately, the information retrieved may not necessarily be applicable to the individual case.^10^ Thus, thyroid cancer survivors have called for the development of a plain-language educational resource, called a decision aid (DA), relating to the decision to accept or reject RRA.^10^ The availability of a web-based DA, with the capacity to retrieve printed material, has been favoured by thyroid cancer survivors.^10^

**Table 1:** Some information needs related to consideration of radioactive iodine remnant ablation (RRA) (or no ablation), as identified by thyroid cancer survivors

Information concepts of interest to patients*	Examples of specific types of information
Disease prognosis (specific to pathologic stage of disease)	General information on disease-related mortality and recurrence risks (some patients preferring numerical data, others preferring non-numerical general concepts) according to individual risk status (i.e. pathologic stage of disease and other factors)
Clearly delineated treatment options	A clear presentation of the choice of RRA or no RRAInformation about the disease-follow-up implications with each choiceA description of the treatment procedure of RRA (with information on preparation and post-treatment precautions)
Treatment rationale	An explanation of the rationale for or against RRA (as applied to the individual’s case)
The potential benefits and risks of treatment options	An explanation of evidence relating to the effect of RRA (or no RRA) on long-term thyroid cancer outcomes (such as recurrence or mortality risk), and any uncertainties of the medical evidence (such as lack of long-term randomized controlled trial data or conflicting results of studies), with information specific for risk status (i.e. pathologic stage of disease)An explanation of radioactive iodine treatment-related risks, including potential short-term side effects or long-term risks. A particular area of interest identified by patients is an explanation of the potential risk of second primary cancers after radioactive iodine treatment.
Current clinical guideline recommendations	An explanation of current clinical practice guideline recommendations, as applied to the individual case
Reproductive considerations (if relevant to the individual patient)	If relevant to the individual, an explanation of radioactive iodine treatment-related reproductive considerations for the immediate and long-term future (including any reassurances to long-term reproductive outcomes, if applicable)

* Table adapted from results of a qualitative study of thyroid cancer survivors (reference 10). The level of detail desired by individual patients relating to each of the concepts is variable, and counselling should be tailored to the individual patient’s preference.

## The use of DAs in medical decision-making

Decision aids are tools or instruments used to inform patients about available treatment options, including evidence about potential benefits and risks of interventions.^11^ DAs facilitate evidence-based patient choice^12^–^14^ and are useful in the clinical setting when there is more than one treatment option.^15^ In a recent systematic review, DAs were reported to improve patient knowledge, result in more accurate risk perceptions, increase the proportion of people active in decision-making and reduce decisional conflict, when compared to usual care.^16^ Also, in another systematic review, cancer-related DAs increased patients’ knowledge compared to usual practice, without increasing anxiety.^17^ However, DAs relating to thyroid cancer care have been lacking.

## The development of a DA on consideration of RRA (or no RRA) in early stage PTC

We recently developed a patient-directed computerized DA explaining the decision to accept or reject RRA for treatment of early stage PTC.^18^ This DA is intended to be used as an adjunct to individualized physician counselling. This DA explains the following concepts relating to RRA decision-making in early stage PTC: disease prognosis (including disease-related mortality and recurrence risks), the rationale (for or against RRA), the potential risks and benefits, the medical evidence uncertainty, the disease-follow-up implications and reproductive considerations.^18^ This DA has been developed in plain English language by a multidisciplinary team, based on reviews of the best available medical evidence.^18^ Furthermore, the DA provides basic information, with the option available for patients to retrieve supplemental detailed information, including numerical data and references, if desired.^18^ Web links to current thyroid cancer clinical practice guidelines and support groups are also provided in the DA programme. The content of the DA is amenable to printing. We have previously reported that a preliminary version of our DA was generally acceptable to lay individuals, thyroid cancer survivors and endocrinologists, although some minor revisions relating to format and wording were suggested.^18^ The preliminary version of the DA was revised, in keeping with suggestions of participants in the initial usability study, and testing of the revised version in a patient with thyroid cancer population is described herein.

## Testing of a thyroid cancer DA on the use of RRA in patients with early stage PTC

Our objectives in the current study were to determine whether our revised DA on RRA increases medical knowledge in patients recently diagnosed with early stage PTC. We also examined the usability of the DA in this population. We recruited individuals aged 18 years or older who had a thyroidectomy since September 1, 2007 and whose surgical pathology reports confirmed the presence of the following TNM stage of PTC: pT1a (multifocal if <1 cm), pT1b, or pT2, N0 (or Nx), M0 (or Mx) (TNM stage, AJCC VII^19^), with no tall cell features (i.e. primary tumours were 4 cm or less in diameter with no known positive lymph nodes and no known distant metastases). Individuals with a single intra-thyroidal papillary thyroid cancer measuring less than one centimetre in diameter or those who had undergone only a hemithyroidectomy (with no completion thyroidectomy) were not eligible for the study because such patients are typically not offered RRA. All participants received usual counselling from their treating physicians and were eligible whether or not they had received RRA. All participants were required to be fluent in written and spoken English and able to read a computer screen. Participants were required to be taking thyroid hormone treatment at the time of testing. The study was approved by the University Health Network Research Ethics Board, and all participants provided informed consent.

### Study procedure and statistical analyses

Participants were tested between November of 2009 and February of 2010. Demographic data were also collected from all participants. Participants self-navigated the DA website on a PC desktop computer (using a secure website) in a research office. Medical knowledge of participants was evaluated before and after DA exposure using a self-administered medical knowledge questionnaire.^18^ The medical knowledge questionnaire includes 10 questions (true or false) on the following topics: early stage PTC prognosis, RRA treatment preparation and treatment procedure, potential radioactive iodine treatment side effects, medical follow-up implications of RRA or no RRA and current medical evidence on the impact of RRA treatment on long-term thyroid cancer outcomes (scored by number of correct responses out of 10, with a maximal score of 10) (details of the development and validation of this questionnaire available from the corresponding author by request).^18^ Usability of the DA was assessed by participants’ responses to an adapted System Usability Scale (SUS) questionnaire for human–computer interaction.^18^,^20^ The adapted SUS questionnaire includes 10 questions on the ease of use, format and applicability of the DA (each question scored on a Likert scale of 1–5 with 1 = strongly disagree to 5 = strongly agree).^18^

Descriptive data were presented as mean and standard deviation (for continuous data) or number and percentage for categorical data. A paired Student’s *t*-test was used to compare scores on the medical knowledge questionnaire before and after exposure to the DA. Unpaired Student’s *t*-tests were used to compare medical knowledge questionnaire scores pre-DA for those who had previously received RRA compared with those who did not. The criterion for statistical significance was set at alpha = 0·05. Quantitative statistical analyses were performed using PASW Statistics 18.0 (IBM, Chicago, IL, USA).

### Results of testing the DA in patients with early stage PTC

The demographic characteristics of participants are shown in Table 2. As expected for PTC, women comprised the largest percentage of participants (41/50, 82%). Most participants had a college or university or higher level education, and most described themselves as very comfortable with computers (Table 1). The mean time spent by participants in reviewing the website was 33 min (minimum 14, maximum 60 min). The mean score on the knowledge questionnaire (number of correct responses out of a maximum score of 10) was 8·04 [standard deviation (SD) 1·43, *n* = 50], prior to exposure to the DA. The mean score on the knowledge questionnaire post-DA was 9·42 (SD 0·88). The DA significantly improved medical knowledge of patients (mean score increase of 1·38 out of 10, SD 1·37) [*t* = 7·13, *P* < 0·001; degrees of freedom (df) = 49]. On the adapted SUS questionnaire, participants generally strongly agreed (mean agreement scores of >4·5 out of 5) with statement that the DA was easy to learn to navigate and use (including printing information, or retrieving supplemental information), that they felt confident using it, and that they believed it would be used frequently by other patients. Participants generally strongly disagreed (mean agreement scores <2 out of 5) with the statements that the DA was unnecessarily complex, cumbersome, inconsistent, or would require technical support.

**Table 2:** Demographic characteristics of participants testing the thyroid cancer decision aid

Characteristic	*N* (%)
Age (years)	
18–30	5/50 (10·0)
31–40	11/50 (22·0)
41–50	18/50 (36·0)
51–60	12/50 (24·0)
61 or older	4/50 (8·0)
Female gender	41/50 (82·0)
Pathologic stage of disease (AJCC VI, TNM classification)*	
T1	23/50 (46·0)
T2	27/50 (54)
N0	37/50 (74)
Nx	13/60 (26·0)
M0	50/50 (100)
Highest level of education	
High school	6/50 (12·0)
College or University	29/50 (58·0)
Postgraduate or professional degree	15/50 (30·0)
Frequency of computer use	
Most days	46/50 (92·0)
Few times per week	3/50 (6·0)
Less than once a month	1/50 (2·0)
Comfort level with computers	
Very comfortable	37/50 (74·0)
Somewhat comfortable	8/50 (16·0)
Comfortable	4/50 (8·0)
Uncomfortable	1/50 (2·0)

* Pathologic stage classified using the American Joint Committee on Cancer Staging Manual, 7th edition (reference 19).

## Discussion

In conclusion, the information needs of patients with early stage papillary thyroid carcinoma considering radioactive iodine remnant ablation are complex. We have found that a patient-directed computerized decision aid, explaining the options of radioactive iodine remnant ablation or no radioactive iodine remnant ablation for early stage papillary thyroid carcinoma, improves patients’ medical knowledge after physician counselling. Furthermore, the web-based format of the decision aid utilized in this study is acceptable to patients. The decision aid tested herein was designed to be used as an adjunct to individualized physician counselling, to meet the information needs of patients who may desire supplemental web-based information. A limitation of the study was that most participants were comfortable with using computers and well-educated, so the findings may not be generalizable to patients unfamiliar with computer technology. However, ultimately, the target population for use of our decision aid are individuals seeking supplemental web-based information sources, so we expect that such individuals would be comfortable using computers. Another important limitation of our study is that for most participants, the decision to accept or reject radioactive iodine remnant ablation had already been made prior to exposure to the decision aid, so we could not examine the impact of the decision aid on the decision-making process (including related psychosocial stress and cultural factors), nor the ultimate decision. However, this was a pilot study, utilized in the planning of a current randomized controlled trial, in which we are comparing the medical knowledge of early stage papillary thyroid carcinoma patients randomized to the decision aid (with usual care) to compared to usual care alone.^21^ In this trial, we are also examining the impact of the decision aid on the final decision, its rationale and satisfaction of patients and physicians.^21^ In the future, thyroid cancer treatment decision aids may become a valuable adjunct in patient education, used to complement physician counselling.
